# Pneumonia and influenza hospitalizations among children under 5 years of age in Suzhou, China, 2005–2011

**DOI:** 10.1111/irv.12405

**Published:** 2016-08-08

**Authors:** Xiyan Zhang, Jun Zhang, Liling Chen, Luzhao Feng, Hongjie Yu, Genming Zhao, Tao Zhang

**Affiliations:** ^1^ Department of EpidemiologySchool of Public HealthFudan UniversityShanghaiChina; ^2^ Key Laboratory of Public Health SafetyMinistry of EducationShanghaiChina; ^3^ Collaborative Innovation Center of Social Risks Governance in HealthShanghaiChina; ^4^ Suzhou Center for Disease Prevention and ControlSuzhouChina; ^5^ Division of Infectious DiseaseChinese Center for Disease Control and PreventionBeijingChina

**Keywords:** children, China, hospitalization, pneumonia, influenza

## Abstract

**Background:**

The disease burden of influenza among children in China has not been well described.

**Objective:**

To estimate the influenza‐associated excess hospitalization rate and compare the hospitalization length and costs between pneumonia and influenza (P&I) and other community‐acquired diseases (CAD) in Suzhou, China.

**Methods:**

We retrospectively collected hospital discharge data on pediatric patients' discharge diagnosis, hospital costs, and length of hospital stay in Suzhou. P&I hospitalization was defined as a primary discharge diagnosis of pneumonia and influenza disease (ICD‐10 codes J09–J18). Other CAD were common community‐acquired diseases among children. Negative binomial regression models were used to estimate the weekly P&I hospitalizations in Suzhou. Excess P&I hospitalizations due to influenza were calculated as the difference in P&I hospitalizations between the epidemic period and the baseline period. Baseline was defined as when the influenza‐positive rates were <5% for two consecutive weeks.

**Results:**

From October 2005 to September 2011, we identified a total of 180 091 all‐cause hospitalizations among children <5 years of age in Suzhou City. The rates of P&I and influenza‐associated excess hospitalizations were highest in the 2009–2010 pandemic and 2010–2011 post‐pandemic seasons. Infants <6 months of age had the highest P&I hospitalization rates, the longest hospital stays (7.5–8.0 days), and the highest hospitalization costs for P&I. Compared with other CAD, children admitted for P&I had longer hospital stays and higher hospitalization costs.

**Conclusions:**

The influenza‐associated P&I hospitalization rates and economic burden were high among children. Targeted influenza prevention and control strategies for young children in Suzhou may reduce the influenza‐associated hospitalizations in this age group.

## 1 Introduction

Influenza is responsible for substantial morbidity and mortality worldwide every year. Estimating influenza‐associated mortality is important for understanding the epidemiology of influenza. However, influenza‐related pediatric deaths are not common,^1^, ^2^ although the substantial number of influenza‐associated hospitalizations among children each year leads to significant burden and economic cost to their families.^3^, ^4^, ^5^, ^6^, ^7^, ^8^, ^9^, ^10^, ^11^, ^12^

Previous prospective studies have estimated high rates of influenza‐associated hospitalization among children from China and other countries.^4^, ^10^, ^11^, ^13^ However, the influenza epidemic varies annually, and such studies are resource intensive. Additionally, when specimens are not collected properly (such as when they are not collected at the right time period of disease course or at the right sites), studies will underestimate the hospitalization rates associated with influenza. Thus, many researchers use statistical models based on morbidity from pneumonia and influenza (P&I) hospitalizations to estimate the number of hospitalizations related to influenza.^2^, ^9^, ^14^, ^15^

Pneumonia and influenza are clinically diagnosed respiratory infectious diseases. Although influenza is not routinely diagnosed in hospitals, it is a vaccine‐preventable disease. Some studies have shown that P&I‐related diagnoses can be used to indirectly estimate the influenza virus activity and the disease burden caused by influenza virus.^9^, ^16^ With the development of electronic health information systems, more researchers are using P&I discharge diagnoses to identify the influenza‐related hospitalizations. Furthermore, identifying P&I hospitalizations using the international code of disease allows for comparison with other studies.

Pneumonia and influenza hospitalizations can impose the substantial socioeconomic burden on families and society. It is estimated that in the United States, the direct costs related to hospitalization due to influenza among children less than 5 years average $809.1 million annually.^17^ In addition to the direct costs of medical care, influenza in this age group has also significant indirect costs, especially in terms of parental work loss while caring for the sick child.^18^ However, in China, data on the influenza‐associated P&I hospitalization rates, especially regarding the economic burden of influenza‐related P&I hospitalization among children, are scarce.^2^, ^19^ The limited data on the influenza disease burden present an obstacle for developing influenza control policies. To address this gap, we collected pediatric discharge data from hospitals, employed a negative binomial regression model to estimate the influenza‐associated P&I hospitalizations among children under 5 years of age between 2005 and 2011 in Suzhou. To better understand the characteristics and economic burden of P&I hospitalization, we compared the hospitalization stays and costs of P&I hospitalizations with those of other common community‐acquired diseases among children.

## 2 Methods

### 2.1 Study site

This study was conducted in Suzhou, a major city located in the southeast area of Jiangsu Province in eastern China. Based on 2010 estimates in Suzhou, the population of children <5 years old comprised 209 276 Suzhou residents and 256 719 migrants (data from the immunization program database). Suzhou consists of five municipal districts and 5 county‐level cities. In 2011, there were 181 hospitals that admitted inpatients, and 36 had over 100 pediatric inpatients in Suzhou city.

### 2.2 Data collection

We selected at least one hospital that admitted pediatric patients from each county and from the municipal districts to participate in this study (Figure S1). The selected hospitals were among the largest hospitals in the county and the districts and admitted at least 2000 pediatric cases per year. We retrospectively collected discharge records for all children less than 5 years old who were admitted to the selected hospitals from 2005 to 2011. We exported the following variables from the electronic health information system (HIS) database of each hospital: admission number, admission date, discharge date, gender, date of birth, discharge diagnosis and/or ICD‐10 codes, and total hospitalization costs (including nursing, physician services, room cost and supplies, diagnostics, and therapeutics).

Pneumonia and influenza hospitalization was defined as a primary discharge diagnosis of pneumonia and influenza disease (ICD‐10 codes J09–J18). The other community‐acquired diseases (CAD) included the following common diseases among children, which represented more than 100 cases per year: upper respiratory tract infection (J06), acute laryngotracheitis and laryngitis (J04), herpes pharyngeal inflammation (B00), bronchitis (J40), hand‐foot‐and‐mouth disease (B08), rotavirus enteritis (A08), acute gastroenteritis (K52.908), infantile enteritis (K52.919), and others.

The annual populations of the following age groups (0 < 6 months, 6 months < 2 years, 2 years < 5 years) were obtained from the immunization program database of the Suzhou Centers for Disease Control and Prevention (CDC), which covers all of the residents and over 95% of the migrant population in these age groups.

Influenza sentinel site surveillance was initiated in 2009 in Suzhou; the influenza virological surveillance data in Suzhou were not available from 2005 to 2009. The influenza virus epidemic patterns of Shanghai, Jiangsu, and Zhejiang are similar to those in Suzhou, in which the influenza virus circulates year‐round with two seasonal peaks. Thus, we collected the influenza virological surveillance data from the areas surrounding Suzhou (Shanghai, Jiangsu, and Zhejiang) from a national surveillance network of influenza‐like illness.^20^ An influenza type or subtype was considered dominant during an influenza season when it accounted for at least 50% of the respiratory specimens that were typed. According to the virological surveillance data, we defined the beginning of the influenza season as October 1.

### 2.3 Ethics statement

The study was approved by the Institute Review Board (IRB) of the School of Public Health, Fudan University. As this was a retrospective, electronic medical data abstraction study that did not collect any personal identifiers or involve any patient contact, this study was exempt from obtaining informed consent.

### 2.4 Statistical analysis

Data analyses were performed using SAS software version 9.0 (SAS Institute Inc., North Carolina, US). Descriptive statistics were used to summarize the continuous variables and discrete variables. Categorical variables were presented as numbers or percentages. Continuous variables were presented as the mean and standard deviation (S.D.), the mean and 95% confidence interval (CI), or the median with interquartile range (IQR). Chi‐square test was used to compare the population characteristics and the P&I hospitalization rates between groups. Kruskal–Wallis test and Mann–Whitney test were used to analyze the length of hospital stay and the hospitalization costs.

### 2.5 Estimating influenza‐associated P&I hospitalization rates

We used negative binomial regression model to estimate the weekly counts of P&I hospitalizations.^2^, ^19^, ^21^ The negative binomial regression models we used were as follows:

$$\begin{array}{cc} Yt_{i}\;=\; & \alpha \exp\;\{\beta_{0}+\beta_{1}\left[t_{i}\right]+\beta_{2}\left[{t_{i}}^{2}\right]+\beta_{3}\left[\sin\left(2\pi t_{i}\right)/52.14\right]\\ & +\beta_{4}\left[\cos\left(2\pi t_{i}\right)/52.14\right]+\beta_{5}\left[\sin\left(2\pi t_{i}\right)/26.07\right]+\beta_{6}\left[\cos\left(2\pi t_{i}\right)/26.07\right]\\ & +\beta_{7}\left[A\left(H1N1\right)t_{i}\right]+\beta_{8}\left[A\left(H3N2\right)t_{i}\right]+\beta_{9}\left[Bt_{i}\right]+\beta_{10}\left[\left(H1N1\text{pdm}\right)t_{i}\right]\}, \end{array}$$

where Y*t*_*i*_ represents the *t*_*i*_ week counts of P&I hospitalizations from 2005 to 2011 for a specific age group. The term “α” is the population offset (α = logpop). The term “*t*_*i*_” is the number of weeks in a series from the beginning of October 2005 through the end of September 2011. We estimated the following β coefficients: β_0_ was the intercept, β_1_ accounted for seasonal changes in P&I hospitalizations, β_2_ accounted for nonlinear time trends, β_3_, β_4_, β_5_, and β_6_ accounted for seasonal changes in P&I hospitalizations (full year and half year), and β_7_, β_8_, β_9_, and β_10_ were coefficients associated with the percentages of specimens testing positive for specific influenza viruses in a given week. The goodness of model fit was tested by *R* ^2^ (1‐SS_reg_/SS_tot_). *R* ^2^ was above 95% for each age group of children.

The P&I hospitalization rates were calculated as the cumulative counts of P&I hospitalizations for each season divided by the corresponding population derived from the immunization database.

### 2.6 Estimating the influenza excess P&I hospitalization rate

For each age group (0 < 6 months, 6 months < 2 years, 2 years <5 years), excess P&I hospitalizations were calculated as the difference in cumulative counts of P&I hospitalizations between the epidemic period and the baseline period for each season. Additionally, the excess P&I hospitalization rates were calculated as the number of excess P&I hospitalizations divided by the population. We defined baselines as the periods when the influenza virus‐positive rates in the national ILI surveillance system for Suzhou and the surrounding areas (including Shanghai, Jiangsu, and Zhejiang) were below 5% for two consecutive weeks.

## 3 Results

In total, we selected nine hospitals for inclusion in this study (Figure S1). In 2011, pediatric admissions from these nine hospitals accounted for 57.3% of all pediatric admissions in Suzhou. Of the selected hospitals, the discharged disease spectrums were similar; approximately 30%–50% of the admissions were P&I cases and 12%–18% were other CAD cases.

From October 2005 to September 2011, there were a total of 180 091 all‐cause hospitalizations among children less than 5 years old in the selected hospitals. Of these, 69 952 (38.8%) were hospitalized due to P&I. The male‐to‐female ratio was similar among P&I and other CAD cases (range 1.4–1.6, *P *> .05). There were more young infants <6 months admitted as P&I than as other CAD (*P *< .001). Additionally, 45 (0.1%) of the 69 952 P&I cases died. The total number of both P&I and other CAD cases increased annually during the study period (*P *< .001) (Table 1).

**Table 1:** The population profile of the included pediatric inpatients among children under 5 years from 2005 to 2011 in Suzhou, China

Characteristics	P&I		Other CAD		All causes	
	N	%	N	%	N	%
Sex						
Male	43 040	61.5	18 450	61.8	112 099	62.3
Female	26 912	38.5	11 399	38.2	67 992	37.7
Age						
0 mo < 6 mo	17 446	24.9	2 769	9.3	49 640	27.6
6 mo < 2 y	31 587	45.2	14 948	50.1	70 437	39.1
2 y < 5 y	20 919	29.9	12 132	40.6	60 014	33.3
Prognosis						
Death	45	0.1	3	0.0	383	0.2
Improved	51 111	73.1	19 197	64.3	113 344	62.9
Cured	17 583	25.1	10 246	34.3	58 879	32.7
Other	1213	1.7	403	1.4	7485	4.2
Season						
2005–2006	5100	7.3	2063	6.9	13 677	7.6
2006–2007	8537	12.2	4928	16.5	23 816	13.2
2007–2008	12 601	18.0	5470	18.3	31 226	17.3
2008–2009	13 324	19.1	5513	18.5	34 383	19.1
2009–2010	14 832	21.2	5859	19.6	37 173	20.7
2010–2011	15 558	22.2	6016	20.2	39 816	22.1
Total	69 952	100.0	29 849	100.0	180 091	100.0

### 3.1 Influenza‐associated P&I hospitalizations

The estimated number of influenza‐related P&I hospitalizations among children less than 5 years old was 5438.7 in the 2005–2006 season and increased to 15483.1 in the 2010–2011 season. The highest estimation of P&I hospitalizations was 15657.7 in the 2009–2010 pandemic season. The number of P&I hospitalizations estimated by the negative binomial regression model was very close to the observed numbers for most of the seasons. However, in the 2007–2008 season when influenza B was circulating, the observed number was higher than that estimated by the model (Figure S2).

The estimated rates of P&I hospitalization were the highest in the 2009–2010 pandemic season and the 2010–2011 post‐pandemic season. Infants <6 months old had the highest rates of P&I hospitalization among children <5 years old, and this rate reached a peak of 66.5 per 1000 children in the 2010–2011 post‐pandemic season (*P *< .01). Older children had lower P&I hospitalization rates (*P *< .01). The P&I hospitalization rates among children 2 < 5 years of age were only a tenth to one‐third of the rates among infants <6 months (Table 2).

**Table 2:** Estimated Influenza‐associated pneumonia and influenza hospitalizations among children <5 years of age in Suzhou, 2005 to 2011

	Estimated influenza‐associated P&I hospitalization(95% CI)				Estimated influenza‐associated P&I hospitalization rate per 1000 person‐year(95% CI)			
	0 mo < 6 mo	6 mo < 2 y	2 y < 5 y	0 mo < 5 y	0 mo < 6 mo	6 mo < 2 y	2 y < 5 y	0 mo < 5 y
2005–2006	2032.9 (1814.5–2251.3)	2347.6 (2244.3–2450.9)	1058.2 (1006.6–1109.9)	5438.7 (5142.4–5735.1)	40.1 (35.8–44.4)	14.2 (13.6–14.9)	4.6 (4.4–4.8)	12.2 (11.5–12.9)
2006–2007	2217.9 (2013.2–2422.5)	3800.5 (3633.7–3967.3)	2269.2 (2177.4––2361.1)	8287.6 (7893.4––8681.7)	42.5 (38.5––46.4)	24.3 (23.2–25.4)	10.9 (10.5–11.3)	19.9 (18.9–20.8)
2007–2008	2710.3 (2413.5–3007.0)	5382.0 (5092.4–5671.6)	3451.8 (3320.5–3583.1)	11544.1 (10913.3–12174.8)	50.7 (45.2–56.3)	32.5 (30.7–34.2)	14.5 (13.9–15.0)	25.2 (23.8–26.6)
2008–2009	2993.7 (2664.9–3322.5)	6338.5 (6022.9–6654.0)	4579.5 (4353.3–4805.7)	13911.7 (13229.0–14594.4)	55.0 (48.9–61.0)	34.7 (32.9–36.4)	16.9 (16.1–17.8)	27.4 (26.1–28.7)
2009–2010	3542.1 (3114.8–3969.4)	7078.3 (6660.0–7496.5)	5037.3 (4737.3–5337.2)	15657.7 (14745.6–16569.6)	65.7 (57.7–73.6)	40.0 (37.6–42.4)	18.8 (17.7–19.9)	31.4 (29.6–33.2)
2010–2011	3854.6 (3447.6–4261.5)	6792.6 (6414.4–7170.8)	4835.9 (4423.0–5248.8)	15483.1 (14528.4–16437.8)	66.5 (59.5–73.5)	39.0 (36.9–41.2)	18.7 (17.1–20.3)	31.5 (29.6–33.5)
2005–2011	17351.5 (16214.1–18488.7)	31739.5 (30798.2–32680.7)	21231.9 (20553.5–21910.4)	70322.9 (67926.0–72719.6)	53.8 (50.2–57.3)	31.1 (30.2–32.0)	14.4 (14.0–14.9)	25.0 (24.1–25.8)

### 3.2 Influenza excess P&I hospitalizations

According to the influenza virological data, there were two influenza epidemic periods per year in Suzhou. The first epidemic period usually started in October and ended in April or May. The second epidemic period varied annually, starting in June, July, or August and lasting for one to three months (Figure S3). The highest influenza‐positive rate was identified in the 2008–2009 influenza season, when seasonal influenza A (H3N2) was identified as the predominant type of influenza virus (Table S1). The annual excess rates of P&I hospitalizations due to influenza among those younger than 5 years old were highest in the 2009–2010 pandemic influenza season (9.6 per 1000 children). Children aged <6 months had the highest excess P&I hospitalization rate, ranging from 5.1 to 26.9 per 1000 children during the study period compared with 1.1 to 4.1 per 1000 children among those aged 2 < 5 years (Table 3).

**Table 3:** Influenza‐associated excess pneumonia and influenza hospitalizations among children <5 years of age in Suzhou, 2005 to 2011

	Predominant type or subtype^a^	Excess number of P&I hospitalizations per year(95% CI)				Excess P&I hospitalization rate per 1000 person‐year (95% CI)			
		0 mo < 6 mo	6 mo < 2 y	2 y < 5 y	0 mo < 5 y	0 mo < 6 mo	6 mo < 2 y	2 y < 5 y	0 mo< 5 y
2005–2006	A/H1N1	728.0 (338.0 to 1118.0)	213.2 (15.6 to 416.0)	−124.8 (−223.6 to 26.0)	816.4 (270.4 to 1372.8)	14.6 (6.8 to 22.4)	1.2 (0.1 to 2.4)	−0.5 (−0.8 to 0.1)	1.7 (0.6 to 2.8)
2006–2007	A/H3N2	332.8 (−67.6 to 438.0)	613.6 (322.4 to 904.8)	109.2 (−78.0 to 291.2)	1060.8 (317.2 to 1799.2)	6.5 (−1.3 to 8.5)	3.9 (2.1 to 5.8)	0.6 (−0.4 to 1.5)	2.6 (0.8 to 4.5)
2007–2008	B	551.2 (−124.8 to 1222.0)	660.4 (10.4 to 1305.2)	270.4 (−26.0 to 572.0)	1476.8 (67.6 to 2891.2)	10.4 (−2.4 to 23.1)	4.2 (0.1 to 8.4)	1.2 (−0.1 to 2.6)	3.4 (0.2 to 6.7)
2008–2009	A/H3N2	317.2 (−348.4 to 982.8)	265.2 (−374.4 to 910.0)	712.4 (301.6 to 1128.4)	1300.0 (−46.8 to 2646.8)	5.9 (−6.5 to 18.2)	1.5 (−2.1 to 5.2)	2.8 (1.2 to 4.4)	2.7 (−0.1 to 5.5)
2009–2010	A/H1N1pdm09, B	1705.6 (940.7 to 2474.9)	1752.9 (1017.3 to 2488.6)	1671.7 (1247.1 to 2096.3)	5132.4 (3858.4 to 6406.4)	31.0 (17.1 to 45.0)	9.2 (5.3 to 13.1)	5.8 (4.4 to 7.3)	9.6 (7.3 to 12.0)
2010–2011	A/H1N1pdm09, A/H3N2	1558.9 (868.4 to 2256.8)	1326.0 (655.2 to 1996.8)	1201.2 (436.8 to 1965.6)	4087.2 (2542.8 to 5636.8)	29.5 (16.4 to 42.7)	8.1 (4.0 to 12.2)	4.8 (1.7 to 7.9)	8.8 (5.5 to 12.1)
2005–2011		5193.7 (1606.3 to 8492.5)	4831.3 (1646.5 to 8021.4)	3840.1 (1657.9 to 6027.5)	13873.6 (7009.6 to 20753.2)	16.4 (5.1 to 26.9)	4.8 (1.6 to 7.9)	2.6 (1.1 to 4.1)	4.9 (2.5 to 7.4)

a Data obtained from the influenza virological surveillance network surrounding the Suzhou area (Shanghai, Jiangsu, and Zhejiang).

### 3.3 Length of hospital stay

The average length of hospitalization due to P&I was 7.1 days during the study period, which was longer than that of other CAD (5.7 days) (*P *< .01) (Table 4). Young infants <6 months had longer hospital stays for P&I than older children aged 2 < 5 years (7.7 days vs 6.6 days, respectively) (*P *< .01). For young infants <6 months, the hospital stays for P&I were longest when the A/H1N1pdm09 and A/H3N2 were circulating in 2010–2011, followed by the 2007–2008 (influenza B) and 2009–2010 (A/H1N1pdm09) seasons. However, for older children aged 2 < 5 years, the longest hospital stays for P&I occurred in the 2008–2009 season when A/H3N2 was circulating. The hospital stays for other CAD were similar for the different age groups of children (*P *> .05).

**Table 4:** Comparison of length of hospitalization stay between P&I and other community‐acquired infections from 2005 to 2011 in Suzhou, China (mean ± SD)

	Predominant type or subtype^a^	Pneumonia and influenza				Other community‐acquired diseases			
		0 mo < 6 mo	6 mo < 2 y	2 y < 5 y	0 mo < 5 y	0 mo < 6 mo	6 mo < 2 y	2 y < 5 y	0 mo < 5 y
2005–2006	A/H1N1	7.5 ± 3.0	7.2 ± 2.5	6.8 ± 2.3	7.3 ± 2.7	5.5 ± 2.8	5.7 ± 2.5	5.6 ± 2.2	5.6 ± 2.5
2006–2007	A/H3N2	7.7 ± 3.4	7.0 ± 2.9	6.5 ± 2.3	7.1 ± 2.9	5.8 ± 2.8	5.2 ± 2.5	5.3 ± 2.2	5.3 ± 2.4
2007–2008	B	7.9 ± 4.0	7.0 ± 2.9	6.6 ± 2.3	7.1 ± 3.1	5.9 ± 3.4	5.5 ± 2.7	5.5 ± 2.4	5.5 ± 2.6
2008–2009	A/H3N2	7.5 ± 3.2	7.0 ± 2.6	7.0 ± 2.3	7.0 ± 2.7	5.8 ± 3.0	5.7 ± 2.5	5.5 ± 2.2	5.6 ± 2.4
2009–2010	A/H1N1pdm09, B	7.8 ± 3.4	7.1 ± 2.8	6.8 ± 2.4	7.1 ± 2.9	6.0 ± 3.4	6.0 ± 2.9	5.8 ± 2.7	5.9 ± 2.9
2010–2011	A/H1N1pdm09, A/H3N2	8.0 ± 4.2	7.1 ± 2.9	6.6 ± 2.5	7.1 ± 3.2	5.7 ± 2.9	6.1 ± 3.4	5.9 ± 3.1	6.0 ± 3.2
2005–2011	/	7.7 ± 3.6	7.1 ± 2.8	6.6 ± 2.4	7.1 ± 2.9	5.8 ± 3.0	5.7 ± 2.8	5.6 ± 2.5	5.7 ± 2.7

Other community‐acquired diseases included the following: upper respiratory tract infection, acute laryngotracheitis and laryngitis, herpes pharyngeal inflammation, bronchitis, hand‐foot‐and‐mouth disease, rotavirus enteritis, acute gastroenteritis, infantile enteritis, and others.

a Data obtained from the influenza virological surveillance network surrounding the Suzhou area (Shanghai, Jiangsu, and Zhejiang).

### 3.4 The hospitalization costs

The median P&I hospitalization cost was 2625.0 Renminbi, the official currency of China (RMB) during our study period, which was higher than that of other CAD hospitalizations (2040.5 RMB) (*P *< .001) (Table 5). Young infants <6 months had higher hospitalization costs for P&I than the older children aged 2 < 5 years (3159.0 RMB vs 2287.2 RMB) (*P *< .01). The hospitalization costs for other CAD increased slightly with age (*P *< .01). For each age group of children, the highest hospitalization costs for both P&I and other CAD occurred in the 2010–2011 season.

**Table 5:** Comparison of hospitalization costs between P&I and other community‐acquired infections from 2005 to 2011 in Suzhou, China (RMB) (median, IQR)

	Predominant type or subtype^a^	Pneumonia and influenza				Other community‐acquired diseases			
		0 mo < 6 mo	6 mo < 2 y	2 y < 5 y	0 mo < 5 y	0 mo < 6 mo	6 mo < 2 y	2 y < 5 y	0 mo < 5 y
2005–2006	A/H1N1	2357.0 (1812.0–3225.5)	2442.0 (1851.0–3246.0)	2401.0 (1799.5–3206.0)	2401.0 (1830.0–3223.0)	1494.0 (1076.0–1951.0)	1629.0 (1212.0–2205.0)	1763.0 (1299.0–2452.0)	1629.5 (1209–2232)
2006–2007	A/H3N2	2773.5 (2099.8–3751.0)	2186.9 (1431.1–3245.8)	1890.8 (1316.3–2896.1)	2295.8 (1515.3–3321.0)	1782.0 (1282.0–2421.5)	1457.5 (888.2–2163.5)	1421.0 (911.9–2326.5)	1487.1 (938.7–2243.0)
2007–2008	B	3192.0 (2437.5–4166.0)	2302.7 (1486.1–3466.2)	2022.8 (1437.4–3010.0)	2470.5 (1605.8–3570.3)	2057.0 (1435.5–2996.8)	1812.5 (1015.8–2649.2)	1811.4 (1074.6–2778.0)	1837.0 (1070.2–2724.0)
2008–2009	A/H3N2	3075.0 (2349.0–4101.0)	2488.0 (1651.0–3637.5)	2229.7 (1572.9–3279.0)	2550.3 (1733.0–3651.0)	2202.0 (1578.9–3071.5)	2260.0 (1380.4–3026.5)	2288.7 (1347.3–3152.7)	2266.0 (1386.5–3080.0)
2009–2010	A/H1N1pdm09, B	3405.8 (2442.8–4583.8)	2659.6 (1733.9–4094.0)	2479.0 (1678.3–3798.6)	2800.0 (1820.0–4156.2)	2325.6 (1599.7–3265.2)	2430.0 (1566.8–3260.3)	2436.3 (1495.6–3287.0)	2420.0 (1544.5–3272.4)
2010–2011	A/H1N1pdm09, A/H3N2	3661.8 (2696.1–4871.4)	2925.6 (1909.1–4285.0)	2577.5 (1756.7–3969.7)	3037.1 (1983.1–4363.6)	2424.4 (1691.5–3345.2)	2538.5 (1584.9–3537.7)	2459.8 (1575.0–3572.7)	2484.5 (1586.0–3542.7)
2005–2011	/	3159.0 (2284.0–4275.0)	2530.1 (1666.0–3756.5)	2287.2 (1574.0–3441.8)	2625.0 (1751.7–3828.0)	2003.4 (1384.0–2880.4)	2032.1 (1240.5–2954.8)	2061.0 (1249.0–3087.9)	2040.5 (1257.9–3006.7)

Other community‐acquired diseases included the following: upper respiratory tract infection, acute laryngotracheitis and laryngitis, herpes pharyngeal inflammation, bronchitis, hand‐foot‐and‐mouth disease, rotavirus enteritis, acute gastroenteritis, infantile enteritis, and others.

a Data obtained from the influenza virological surveillance network surrounding the Suzhou area (Shanghai, Jiangsu, and Zhejiang).

## 4 Discussion

Our findings demonstrate that influenza activity is associated with P&I‐associated hospitalization rates among children less than 5 years old in Suzhou. The estimated P&I hospitalization rates were highest in the 2009–2010 pandemic season and the 2010–2011 post‐pandemic season, when A/H1N1pdm09 was circulating. The average estimated P&I hospitalization rate among children <5 years old was 25 per 1000 children during the study period, which was consistent with studies in eastern 19 and central China.^13^

Among children less than 5 years old, the influenza‐associated P&I hospitalization rates were inversely related to the age of the child, being highest in those younger than 6 months. Although this is consistent with findings from the United States 22 and Finland,^3^ the influenza hospitalization rates estimated from the influenza network data or prospective surveillance have shown the highest influenza hospitalization rates in children 6–24 months in central China.^13^ The main reason for this discrepancy may be that the influenza network data or prospective surveillance only detected influenza virus, and estimations based on laboratory tests may lead to lower estimations of influenza‐associated hospitalization. In contrast, P&I hospitalizations are diagnosed based on clinical characteristics, which can capture almost all of the related hospitalizations. In addition, P&I hospitalizations may include influenza‐ and other respiratory virus‐associated hospitalizations, especially respiratory syncytial virus (RSV), which often targets young infants under 6 months.

The median cost of the P&I hospitalizations during the study period was 2625 RMB (equal to 423 USD with a ratio of 1 USD:6.2 RMB); this cost was a little lower than that found in our previous study conducted with children hospitalized with influenza infection (624 USD),^23^ but much higher than the result of another study from China of 231 USD per influenza hospitalization among children ≤15 years from 2009 to 2011.^24^ These differences are influenced by local economics, as the income and salary are higher in Suzhou than in other areas of China, and the definition of the diseases varies between the studies.

Compared with other age groups, infants under 6 months of age had the longest length of hospital stay and the highest cost of P&I hospitalization. These findings are consistent with our previous study on hospitalizations for influenza infection: Those aged >60 months had shorter hospital stays compared with infants under 6 months of age (OR = 0.45).^23^ The higher hospitalization costs and longer hospital stays in this age group of children present significant burdens to their families. The risk of illness in children less than 6 months of age could potentially be prevented by maternal immunization,^25^ but the influenza vaccine is not approved in China for this population.

Compared with children aged 2 < 5 years old, the children aged 6 < 24 months had higher P&I hospitalization rates and costs. These results were consistent with the findings from a study in central China of the same season.^13^ The influenza vaccine is the most effective method for influenza control and prevention. Although the effectiveness of the vaccine varies annually, the average influenza vaccine effectiveness has been estimated to be approximately 50%–72%.^26^, ^27^ The data on influenza vaccine effectiveness in China are consistent with those of developed countries.^28^, ^29^, ^30^ However, the influenza vaccination coverage among children ≤5 years of age in China was low, ranging from a low of 8.6% to a high of 26.4% between the 2009 and 2012 influenza seasons, with no increasing trends by year.^28^, ^31^ Therefore, children less than 5 years of age, especially children under 24 months, could benefit from increasing rates of influenza vaccination.

This study has several limitations. First, P&I hospitalizations are associated with numerous pathogens in addition to influenza, such as RSV, parainfluenza virus, and adenovirus. However, we did not conduct laboratory testing for respiratory pathogens in our study, and therefore, we cannot specifically estimate the influenza‐associated hospitalizations. Second, the influenza virus surveillance data originated from the surrounding provinces and not specifically Suzhou city, which may suggest that the data did not directly reflect the influenza epidemic status in Suzhou and could cause some bias in estimating the excess P&I hospitalizations among children in Suzhou. Third, although we considered seasonal changes that could represent potential confounding factors, there may still have been some unmeasured factors such as income or distance between residence and hospitals that could have affected the estimates of influenza‐associated hospitalizations for P&I.

Pneumonia and influenza hospitalizations appear to be an increased burden on young children and their families in Suzhou. Children less than 5 years of age, especially children under 24 months, could benefit from increasing the influenza vaccination coverage.

## Funding

This research was supported by the Cooperative Agreement Number, 5U2GGH000018, funded by the Centers for Disease Control and Prevention, and was partially supported by the Shanghai Leading Public Health Discipline Project [12GWZX0101].
