# Supplementary material for: Pneumonia and influenza hospitalizations among children under 5 years of age in Suzhou, China, 2005–2011
Source: Influenza Other Respir Viruses. 2016 Aug 8;11(1):15–22. doi: 10.1111/irv.12405 (PMC5155646; doi:10.1111/irv.12405)
Supplement: Supplementary file 1 [file IRV-11-15-s001.docx]

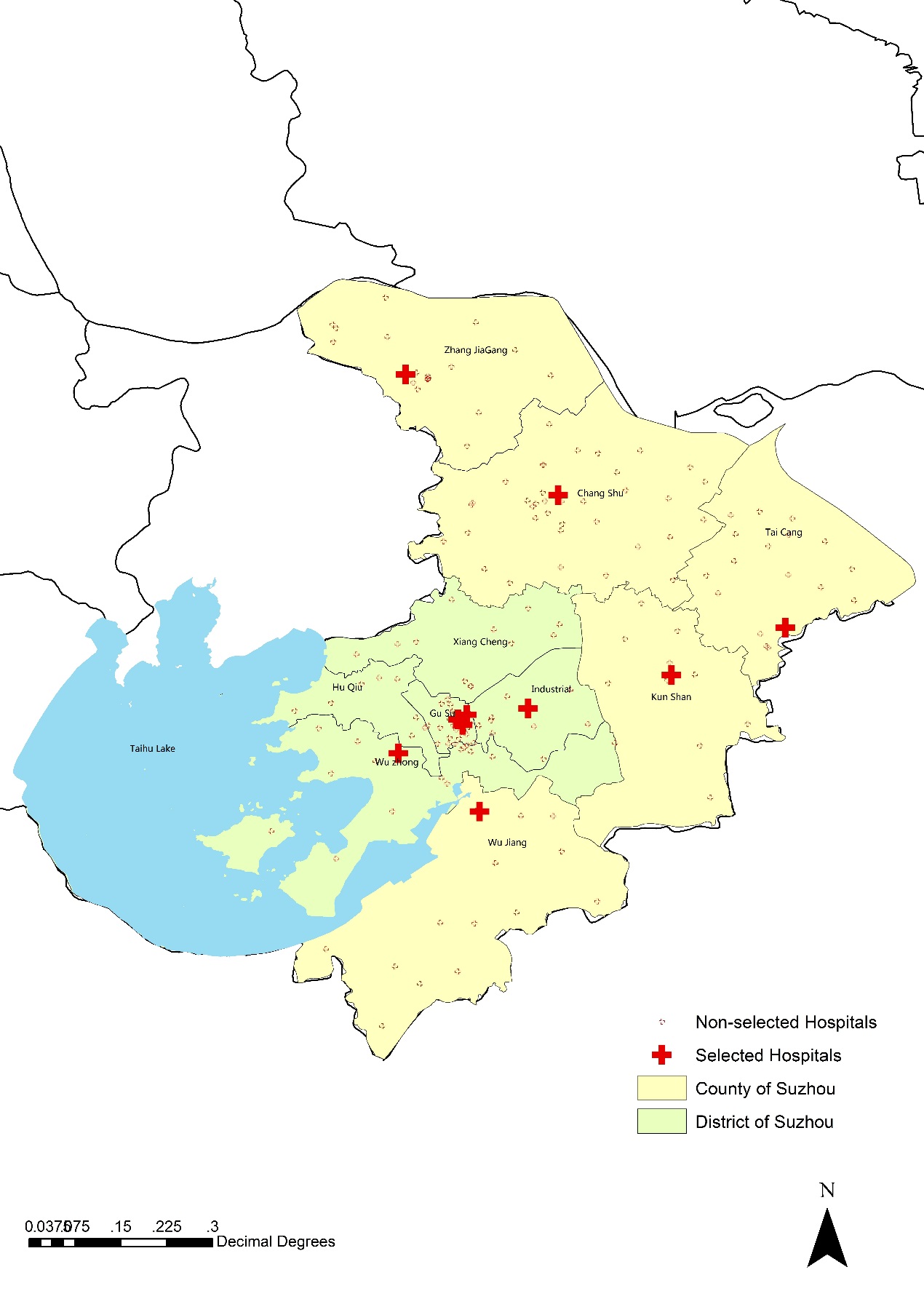


Supplemental figure 1 distribution of hospitals in Suzhou city

Supplemental figure 2 The observed and estimated pneumonia and influenza (P&I) hospitalization numbers among children <5 years old in Suzhou from 2005 to 2011

Supplemental figure 3 The influenza virus epidemic trends in the Suzhou surrounding area from 2005 to 2011
