# Supplementary material for: Pneumonia and influenza hospitalizations among children under 5 years of age in Suzhou, China, 2005–2011
Source: Influenza Other Respir Viruses. 2016 Aug 8;11(1):15–22. doi: 10.1111/irv.12405 (PMC5155646; doi:10.1111/irv.12405)
Supplement: Supplementary file 2 [file IRV-11-15-s002.docx]

Supplemental table1 Influenza laboratory surveillance from 2005-2006 to 2010-2011 influenza seasons in Jiangsu, Zhejiang and Shanghai, China

|  | Total Number of tested specimens | Number of influenza virus positive specimens (%) | | | | | Predominant type or subtype |
| --- | --- | --- | --- | --- | --- | --- | --- |
|  |  | H1N1 | H1N1pdm | H3N2 | B | Total |  |
| 2005-2006 | 1981 | 136(6.9) | 0(0.0) | 12(0.6) | 36(1.8) | 184(9.3) | A/H1N1 |
| 2006-2007 | 2170 | 6(0.3) | 0(0.0) | 161(7.4) | 68(3.1) | 235(10.8) | A/H3N2 |
| 2007-2008 | 1882 | 50(2.7) | 0(0.0) | 63(3.4) | 101(5.4) | 214(11.5) | B |
| 2008-2009 | 4557 | 126(2.8) | 33(0.7) | 689(15.1) | 55(1.2) | 903(19.8) | A/H3N2 |
| 2009-2010 | 12950 | 4(0.0) | 769(5.9) | 298(2.3) | 677(5.2) | 1748(13.4) | A/H1N1pdm09, B |
| 2010-2011 | 9007 | 0(0.0) | 295(3.3) | 301(3.3) | 214(2.4) | 810(9.0) | A/H1N1pdm09, A/H3N2 |
